# Diagnostic accuracy of sonication fluid cultures from prosthetic components in periprosthetic joint infection: an updated diagnostic meta-analysis

**DOI:** 10.1186/s13018-023-03662-3

**Published:** 2023-03-08

**Authors:** Guanrong Peng, Qiang Liu, Zhenhua Guan, Min Liu, Xiaobo Sun, Xingyang Zhu, Jinlun Chen, Wenjun Feng, Jie Li, Jianchun Zeng, Zhangrong Zhong, Yirong Zeng

**Affiliations:** 1 The First Clinical Medical School, Guangzhou University of Chinese Medicine, Jichang Road 12#, District Baiyun, Guangzhou, Guangdong People’s Republic of China; 2 Yudu People’s Hospital, Huancheng North Road 2#, District Yudu, Ganzhou, 342300 Jiangxi People’s Republic of China; 3 Peking University People’s Hospital, Arthritis Clinic and Research Center, Beijing, People’s Republic of China; 4 Department of Orthopaedics, The First Affiliated Hospital of Guangzhou University of Chinese Medicine, Jichang Road 16#, District Baiyun, Guangzhou, 510405 Guangdong People’s Republic of China

**Keywords:** Periprosthetic joint infection, Sonication fluid culture, Periprosthetic tissue culture, Joint arthroplasty, Diagnosis, Diagnostic accuracy, Heterogeneity

## Abstract

**Background:**

Periprosthetic joint infection (PJI) is the most serious complication following total joint arthroplasty (TJA) and has a significant impact on patients and the national healthcare system. To date, the diagnosis of PJI is still confronted with dilemmas. The present study investigated the validity of sonication fluid culture (SFC) for removing implants in the diagnosis of PJI after joint replacement.

**Methods:**

From database establishment to December 2020, relevant literature was retrieved from the PubMed, Web of Science, Embase and Cochrane Library databases. Two reviewers independently performed quality assessment and data extraction to calculate the pooled sensitivity, specificity, positive likelihood ratio (PLR), negative likelihood ratio (NLR), area under the curve (AUC) and diagnostic odds ratio (DOR) to evaluate the diagnostic value of overall SFC for PJI.

**Results:**

A total of 38 eligible studies including 6302 patients were selected in this study. The pooled sensitivity, specificity, PLR, NLR, and DOR of SFC for PJI diagnosis were 0.77 (95% confidence interval [CI], 0.76–0.79), 0.96 (95% CI, 0.95–0.96), 18.68 (95% CI, 11.92–29.28), 0.24 (95% CI, 0.21–0.29), and 85.65 (95% CI, 56.46–129.94), respectively, while the AUC was 0.92.

**Conclusion:**

This meta-analysis showed that SFC was of great value in PJI diagnosis, and the evidence of SFC on PJI was more favorable but not yet strong. Therefore, improvement of the diagnostic accuracy of SFC is still necessary, and the diagnosis of PJI continues to warrant a multiplex approach before and during a revision procedure.

## Introduction

Periprosthetic joint infection (PJI) after primary total joint arthroplasty (TJA) is regarded as a devastating complication [1], with an infection rate ranging from 0.88% to 2.18% [2, 3]. The prevalence of PJI in TJA is increasing as the number of joint replacements grows, leading to significant influences on the patient and the national healthcare system owing to multiple surgeries, prolonged hospital stays, patient suffering, and the high cost of treatments [4, 5]. Currently, the diagnosis of PJI depends on serum inflammatory markers, arthrocentesis-based studies, and intraoperative tissue cultures. However, traditional diagnostic techniques are limited in their capacities to detect low-grade infections caused by bacteria, which exist on the surface of implants in the form of biofilms rather than plankton [6]. Therefore, accurately identifying shielded microbes on the prosthesis surface and their respective sensitivities is a major challenge in the management of periprosthetic joint infections (PJIs).

Synovial fluid and intraoperative periprosthetic tissue cultures are considered to be the standard method for the diagnosis of PJI as defined by the Infectious Diseases Society of America (IDSA), the Musculoskeletal Infection Society (MSIS) and the International Consensus Meeting (ICM) [7–9]. However, the high rate of false negatives and low sensitivity of microbial cultures have attracted the attention of scholars. Studies have reported an unacceptably high false-negative rate for these procedures, ranging from 17 to 53% [10–12]. Trampuz et al. reported a low sensitivity of 61% (95% CI: 49–72) for microbial cultures [13]. In addition, microbial detection of polymicrobial infections by culture is as low as 13% to 17% [14]. Therefore, it is urgent for joint surgeons to find an efficient, inexpensive and convenient diagnostic method for PJI. In recent years, the role of sonication fluid culture (SFC) of the removed implant as a diagnostic tool of PJI continues to evolve. An inherent advantage of sonication is that it can destroy bacteria in biofilms and increase the number of culturable bacterial cells [15]. Several groups have demonstrated that SFC has higher sensitivity than periprosthetic tissue culture in the diagnosis of PJI, especially when antibiotics have been used shortly before explantation [13, 16–19]. In contrast, Van Diek et al. [20] reported that the sensitivity of ultrasound liquid analysis was lower than that of tissue culture, with 0.47 (95% CI: 0.35–0.59) and 0.68 (95% CI: 0.56–0.78), respectively. Currently, some studies have shown that the sensitivities (range 0.47 to 0.93) and specificities (range 0.67 to 1.0) of SFC were inconsistent in evaluating the diagnostic value of PJI [14, 15, 20–52]. To our knowledge, there is no consensus as to the most appropriate tests for excluding PJI after primary TJA.

Therefore, we conducted a systematic review and meta-analysis to synthesize the available evidence on the accuracy of SFC in the diagnosis of PJI and provide further evidence for its clinical application.

## Methods

Our study strictly followed the Preferred Reporting Items for Systematic Reviews and Meta-Analyses (PRISMA) guidelines [53]. This protocol was determined by all the authors. We performed a literature search, screened the studies identified, the data statistics, the results combination, and the manuscript, and evaluated the studies that related to the application of SFC in PJI diagnosis.

### Search strategy

We (Guanrong Peng and Qiang Liu) systematically searched online databases such as PubMed, Cochrane Library, Embase, and Web of Science (from the time of database inception to December 2020) under the guidance of the Cochrane review method. The medical subject headings (MeSH) and keywords were used as follows: “periprosthetic joint infection” or “prosthesis-related infection” or “periprosthetic infection” represents the disease, “ultrasonics” or “sonications” or “sonication” or “ultrasonic” stands for diagnostic method, “sensitive” or “sensitivity and specificity” or “predictive and value” or “predictive value of tests” or “accuracy” represents the research type. Throughout the retrieval process, only studies in English were included. To obtain valuable articles for this study, we also manually searched the reference lists of eligible studies and review articles after database screening. The full database search strategies for our study can be found in “Appendix”.

### Eligibility criteria

**Fig. 1:**
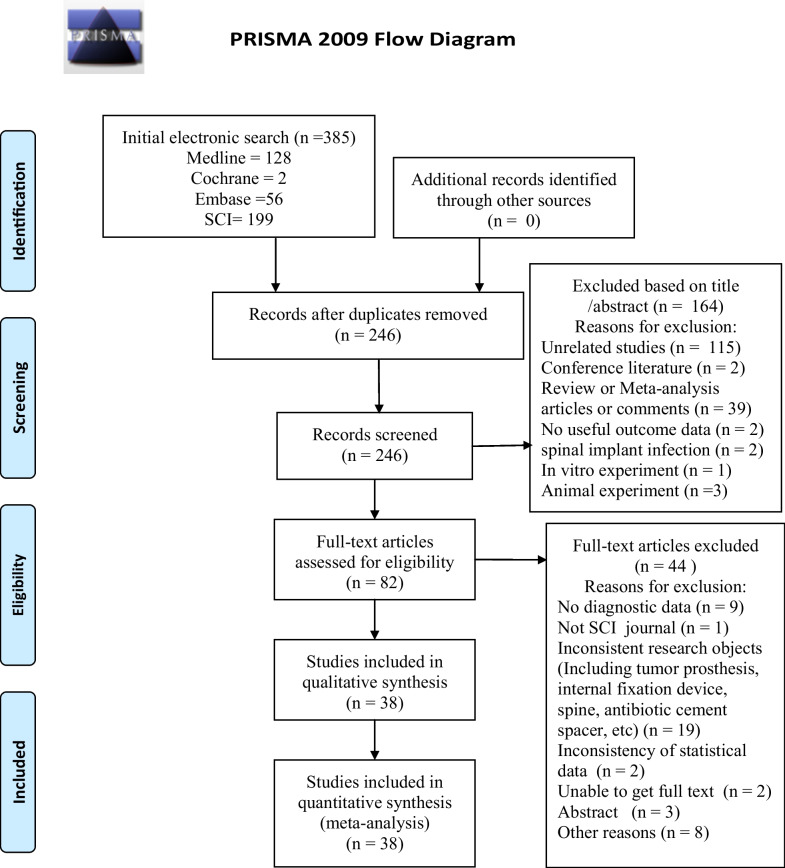
Flow diagram for study selection

We included all studies that reported the accuracy of SFC in the diagnosis of PJI after TJA. Two authors (Guanrong Peng and Qiang Liu) independently scanned the titles, abstracts and full texts sequentially, and eligible studies were included in this systematic review. When there was a disagreement between the two authors regarding inclusion, consensus was reached by consultation with another author (Professor Yirong Zeng). Inclusion criteria that eligible studies had to meet were as follows: (1) focusing on the diagnosis of SFC in PJI; (2) cutoff or range definitions of the tests; (3) reference standard, such as “Musculoskeletal Infection Society (MSIS)”, “International Consensus Meeting (ICM)”, “Infectious Diseases Society of America (IDSA)” or “European Bone and Joint Infection Society (EBJIS)”, and other culture or clinical diagnosis criteria; and (4) providing data (including true positive, false negative, false positive, and true negative) for completion of 2-by-2 tables. Studies lacking sensitivity and specificity values or having duplicate data were excluded (Fig. 1).

### Data extraction and quality assessment

Relevant information was extracted by the two reviewers (Guanrong Peng and Qiang Liu) independently from all selected studies with a standardized data collection form. The following information was abstracted: (1) study baseline characteristics (such as the author name, publication year, country, study design, time of follow-up, number ratio of non-PJI vs. PJI, sex distribution, average age, and joint type, among others); (2) study intervention characteristics (the cutoff value of SFC, diagnostic criteria, etc.); and (3) outcome indicators, including sensitivity and specificity. Then, the true-positive (TP), false-positive (FP), true-negative (TN) and false-negative (FN) data used to construct the 2-by-2 tables were further calculated. Finally, we obtained the positive likelihood ratio (PLR), negative likelihood ratio (NLR), area under the curve (AUC) and diagnostic odds ratio (DOR) of the SFC subgroup analysis for PJI diagnosis.

**Fig. 2:**
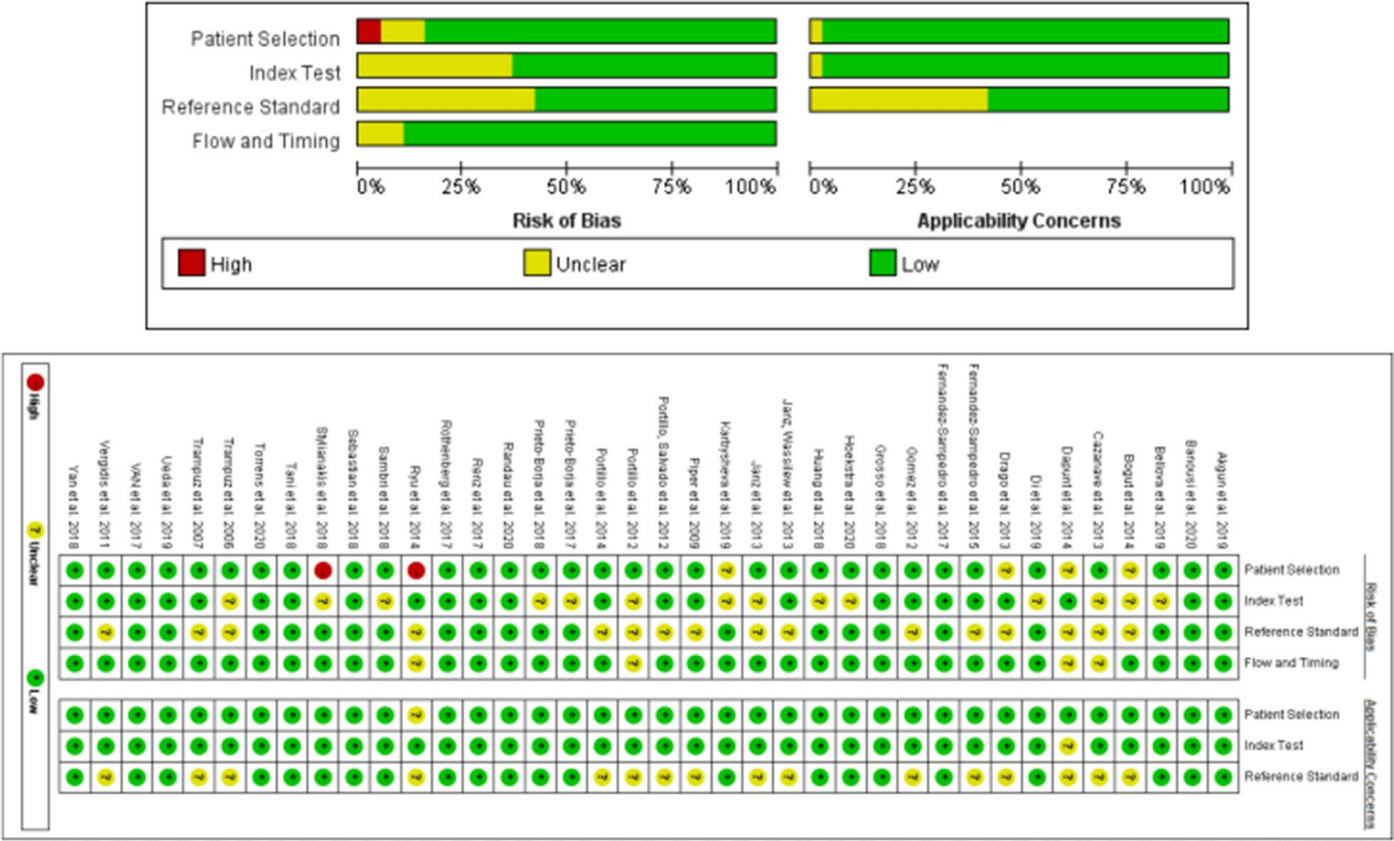
Quality assessment of included studies based on QUADAS-2 tool criteria

The risk of bias and concerns regarding applicability of each included study were independently assessed by utilizing the Quality Assessment of Diagnostic Accuracy Studies-2 (QUADAS-2) tool in Revman (version 5.3) software [54]. QUADAS-2 consists of four key domains: patient selection, index test, reference standard, and flow and timing. All domains of the QUADAS-2 were assessed for risk of bias, and the first three areas were also evaluated for clinical practicability. The risk of bias level was determined as “low”, “high” or “uncertain” according to the answers (“yes”, “no” or “uncertain”) to the relevant landmark questions contained in each domain. When the two authors disagreed, the third author made the final decision. The results of the QUADAS-2 evaluation of the studies included in the meta-analysis are shown in Fig. 2.

### Statistical analysis

Meta-DiSc 1.4 software and Stata 14.0 were used for data analysis and image production. We calculated the values of TP, FP, FN, and TN according to the sensitivity and specificity of each eligible study to construct 2-by-2 tables. The bivariate random effect model was used to calculate the pooled sensitivity, specificity, PLR, NLR, AUC, DOR, and 95% confidence intervals (CI) for each study by using Meta-DiSc software.

Forest plots obtained from the random effect model were used to summarize the results and test the heterogeneity. The heterogeneity among studies was expressed as the inconsistency index (*I*^*2*^) statistic, with values ranging from 0 to 100%. High heterogeneity was defined as *I*^*2*^ values of > 75%; 50% ≤ I2 ≤ 75% indicated moderate heterogeneity and *I*^*2*^ < 50% indicated low heterogeneity. If large heterogeneity was caused by the threshold effect, the Spearman correlation coefficient of the logarithms of sensitivity and 1-specificity was performed to evaluate the threshold effect. The threshold effect was considered to have a significant difference at *p* < 0.05. When there was high heterogeneity, we conducted subgroup analysis to find the potential sources of heterogeneity. The type of study design, the sample type, the publication year, the threshold used in the study, the number of joints, the reference standard, and the country or region were considered to be the variables that may have caused heterogeneity. Then, the heterogeneity caused by the nonthreshold effect was detected by calculating the heterogeneity of the Chi-squared value (Cochran Q) and the inconsistency index (*I*^*2*^) of the DOR of each test. When *I*^*2*^ > 50% or *p*≦0.1, significant heterogeneity was considered among the studies, and we used a random effects model to pool the related effects and perform a subgroup analysis; otherwise, the fixed-effects model was used (*I*^*2*^≦50% or *p* > 0.1). In addition, we drew a summarized receiver operating characteristic curve (SROC) and AUC. The higher values of the AUC indicate more accurate test results of SFC.

Furthermore, sensitivity analysis was performed for each eligible study using the “Midas” command of Stata software. Deeks' funnel plot was used to estimate publication bias, while the Fagan plot clearly displayed the change in diagnostic value of SFC for PJI. We performed the Z-test to compare sensitivity, specificity and value of the AUC among the different subgroups when *p* < 0.05 was considered statistically significant.

## Results

### Study selection

After systematically searching the aforementioned four databases, we initially obtained a total of 385 articles, with 199 from Web of Science, 128 from PubMed, 56 from Embase, and 2 from the Cochrane Library. A total of 139 duplicate studies were removed from these 385 studies, leaving 246 for title and abstract review. A total of 82 studies were eligible for full-text article review after reviewing the titles and abstracts. After reviewing the full text of each study in detail, another 44 studies were excluded. Eventually, a total of 38 studies were included in our systematic review and meta-analysis (Fig. 1).

### Quality assessment

Two authors (Jinlun Chen and Xiaobo Sun) used the QUADAS-2 tool to assess the quality of all included studies. As shown in Fig. 2, two studies showed “high risk” for patient selection, while other studies showed “low risk” or “unclear” for patient selection, reference standard, index test, and flow and timing bias. For the index test and the reference standard bias, 14 (36.8%) and 16 studies (42.1%) showed unclear risk, respectively. The reason for this assessment was to interpret the results of the index test without knowing the results of the reference standard. Most of the studies in this meta-analysis were evaluated as “low risk” in the applicability section, and there was no “high risk”.

### Study characteristics

These 38 studies [13–17, 20–52] included 19 prospective studies, 17 retrospective studies and 2 not described in detail. Among the 38 studies, 8 reported MSIS criteria, 5 reported ICM criteria, 5 reported IDSA criteria, and 20 reported other reference standards. In terms of the diagnostic threshold, 10 studies used 50 colony-forming units (CFU)/ml, 6 used 20 CFU/plate, and the remaining 22 used other values. Twenty-five of the studies were from European countries, 9 were from the USA, and the other 4 were from Asian countries. All studies included in this meta-analysis were selected because they cultured the sonication fluids obtained from the prostheses removed by surgery after ultrasonic treatment. A total of 6302 patients were enrolled in our study, among whom 2290 (36.3%) were confirmed to have PJI. The detailed characteristics of all the studies are shown in Table 1. The data extraction results of each study are summarized in Table 2.

**Table 1:** Characteristics of the studies in meta-analysis for the diagnosis of PJI applying sonication

Study (reference)	Publication year	Country	Study design	Inclusion interval	Number (joints)	NO. of non-PJI/PJI	Gender (M/F)	Median age (non-PJI/PJI)	Sample processing time (after prosthesis removal)	Sample site(s)	Antibiotic use before revision
Hoekstra et al	2020	Netherlands	R	2011–2016	226	139/87	105/121	69/70	Within 4 h	H, K	No
Randau et al	2020	Germany	R	2018 (1–3,10–12)	40	17/23	NA	NA	NA	H, K	No
Torrens et al	2020	Spain	R	2000.10–2016.4	99	68/31	11/51	63/67	Immediately	S	NA
Banousi et al	2020	Greece	P	2014.5–2019.6	234	143/91	111/123	60.6/67.7	Within 6 h	H, K, S, E	Yes
Akgun et al	2019	Germany	R	2014.7–2018.12	72	44/28	33/39	69.9/69.6	Within 1 h	S	No
Bellova et al	2019	Germany	R	2017.3–2018.4	257	112/145	84/173	69.5/70	NA	H, K, S	Yes
Ueda et al	2019	Japan	P	2014.1–2016.1	67	50/17	20/46	67/62	Within 48 h	H, K, E, A	Yes
Karbysheva et al	2019	Germany	P	NA	331	252/79	NA	NA	NA	H, K	NA
Di et al	2019	Italy	R	2016.12–2019.1	50	37/13	18/32	76/76	NA	H, K	NA
Tani et al	2018	Greece	P	2012.7–2016.7	114	53/61	29/85	70/70	Within 6 h	H, K	No
Sebastian et al	2018	India	P	2016.7–2017.6	40	13/27	22/18	55.9/55.8	Within 2 h	H, K	Yes
Yan et al	2018	China	P	2016.3–2017.9	229	125/104	127/102	67/66.5	NA	H, K, S, E	Yes
Grosso et al	2018	USA	P	2010.8–2013.4	53	28/25	28/25	62/62	Immediately	S	No
Sambri et al	2018	Italy	P	2014.4–2016.7	117	73/44	48/70	69/69	Immediately	H, K	No
Prieto et al	2018	Spain	R	2011.1–2014.6	276	95/181	75/125	70.9/70.9	Within 24 h	H, K, S	Yes
Huang et al	2018	China	R	2014.4–2017.4	67	14/53	26/41	62.5/62.5	Within 30 min	H, K	No
Stylianakis et al	2018	Greece	P	2011.9–2015.4	114	87/27	32/82	72.4/70.6	Immediately	H, K	No
Rothenberg et al	2017	USA	R	2012.10–2016.5	503	325/178	245/258	65.6/64	NA	H, K	Yes
Renz et al	2017	Germany	P	2014.12–2015.10	111	33/78	50/61	69/75	Within 6 h	H, K, S, E	No
VAN et al	2017	Netherlands	R	2011.3–2012.11	252	177/75	81/152	67/64	Within 6 h	H, K	No
Prieto et al	2017	Spain	P	2014.5–2015.5	88	50/38	NA	NA	Within 24–48 h	H, K, S	Yes
Fernandez et al	2017	Spain	P	2009.2–2014.2	498	368/130	66/64	NA/64.8	NA	H, K	No
Fernandez et al	2015	Spain	P	2009.2–2011.9	198	174/24	86/112	69.5/68.2	NA	H, K	Yes
Bogut et al	2014	Poland	R	NA	76	54/22	NA	72.3/67.5	Within 2 h	H	Yes
Ryu et al	2014	USA	R	1998.5–2012.7	45	15/30	30/15	66/68.5	Within 6 h	K	NA
Dapunt et al	2014	Germany	NA	NA	77	52/25	NA	NA	NA	H, K, S	NA
Portillo et al	2014	Spain	P	2010.7–2013.7	231	162/69	88/143	76/74	NA	H, K, S, E	Yes
Drago et al	2013	Italy	NA	NA	76	34/42	30/46	68/71	NA	NA	NA
Cazanave et al	2013	USA	R	2006.4–2011.5	434	290/144	207/227	68/66	Within 6 h	H, K	Yes
Janz et al	2013	Germany	P	2010.10–2011.12	102	65/37	NA	NA	Within 6 h	H	No
Janz et al	2013	Germany	P	2010.10–2011.3	59	36/23	26/33	67/67	Within 6 h	H, K	No
Portillo et al	2012	Spain	R	2010.7–2012.4	135	100/35	51/84	73/73	NA	H, K, S, E	Yes
Gomez et al	2012	USA	R	2006.4–2011.5	366	231/135	183/183	66/66	NA	H, K	Yes
Portillo et al	2012	Spain	P	2010.7–2011.7	86	62/24	32/54	73/73	NA	H, K, S, E	Yes
Vergidis et al	2011	USA	R	2007.7–2010.7	36	27/9	7/29	60/61	Within 6 h	E	Yes
Piper et al	2009	USA	R	2004.8–2008.11	134	101/33	59/75	67/60	Within 6 h	S	Yes
Trampuz et al	2007	USA	P	2003.8–2005.12	331	252/79	157/174	70/68	Within 6 h	H, K	Yes
Trampuz et al	2006	USA	P	1998.7–2003.8	78	54/24	42/36	71.5/71	Within 4 h	H, K	Yes

*NA* not available; *P* prospective study; *R* retrospective study; *H* Hip; *K*  Knee; *S* Shoulder; *E* Elbow; *A* Ankle

**Table 2:** Data extracted for the construction of 2 × 2 table

Study (reference)	Publication year	Solution for prosthesis (amt [ml])	Culture period	Centrifugation	Vortexing	Cutoff value	Reference standard	Sen	Spe	TP	FP	FN	TN	Total (joints)
Hoekstra et al	2020	Ringer’s solution	Aerobic and anaerobic for 10 days	No	Yes	NA	ICM	0.81	0.98	70	3	17	136	226
Randau et al	2020	Sterile saline	Aerobic and anaerobic for 14 days	Yes	Yes	NA	MSIS	0.74	0.82	17	3	6	14	40
Torrens et al	2020	Sterile saline (200–400)	Aerobic and anaerobic for 14 days	No	Yes	50 CFU/ml	ICM	0.8	0.93	25	5	6	63	99
Banousi et al	2020	Ringer’s solution (50–200)	Aerobic for 7 days and anaerobic for 14 days	Yes	Yes	50 CFU/ml	IDSA	0.91	1.00	83	0	8	143	234
Akgun et al	2019	Normal saline	Aerobic and anaerobic for 14 days	No	Yes	50 CFU/ml	ICM	0.75	0.82	21	8	7	36	72
Bellova et al	2019	Ringer’s solution (400)	Aerobic for 4 days and anaerobic for 14 days	Yes	Yes	NA	ICM	0.88	0.85	128	17	17	95	257
Ueda et al	2019	Sterile saline (500)	Aerobic for 5 days and anaerobic for 10 days	Yes	Yes	0.1 CFU/ml	MSIS	0.71	1.00	12	0	5	50	67
Karbysheva et al	2019	NA	NA	NA	NA	NA	MSIS	0.79	0.99	62	3	17	249	331
Di et al	2019	NA	NA	NA	NA	NA	ICM	0.84	0.91	11	3	2	34	50
Tani et al	2018	Ringer’s solution (400)	Aerobic for 5 days and anaerobic for 7 days	Yes	Yes	50 CFU/ml	IDSA	0.77	0.98	47	1	14	52	114
Sebastian et al	2018	Normal saline(200–800)	Aerobic for 2–4 days and anaerobic for 7–14 days	Yes	Yes	20 CFU/ml	MSIS	0.93	1.00	25	0	2	13	40
Yan et al	2018	NA	Aerobic and anaerobic for 5–12 days	NA	NA	2 CFU/ml	IDSA	0.73	1.00	76	0	28	125	229
Grosso et al	2018	Ringer’s solution	Aerobic and anaerobic for 12.1 days	No	Yes	20 CFU/ml	IOF, H, M	0.56	0.93	14	2	11	26	53
Sambri et al	2018	Sterile saline	Aerobic for 7 days and anaerobic for 7–14 days	Yes	Yes	NA	MSIS	0.89	0.95	39	4	5	69	117
Prieto et al	2018	Sterile phosphate buffer(50)	Aerobic and anaerobic for 7 days	Yes	No	NA	IDSA	0.70	0.98	127	2	54	93	276
Huang et al	2018	Ringer’s solution (400)	Aerobic for 5 days and anaerobic for 14 days	Yes	Yes	NA	MSIS	0.83	0.86	44	2	9	12	67
Stylianakis et al	2018	Ringer’s solution	Aerobic for 7 days and anaerobic for 14 days	Yes	Yes	NA	IOF, H, M	0.63	0.82	17	16	10	71	114
Rothenberg et al	2017	Ringer’s solution (400)	Aerobic for 4 days and anaerobic for 14 days	Yes	Yes	50 CFU/ml	MSIS	0.90	0.93	160	24	18	301	503
Renz et al	2017	normal saline	Aerobic and anaerobic for 14 days	No	Yes	50 CFU/ml	EBJIS	0.58	1.00	45	0	33	33	111
VAN et al	2017	Ringer’s solution (500–800)	Aerobic and anaerobic for 5 days	Yes	Yes	50 CFU/ml	MSIS	0.47	0.99	35	2	40	175	252
Prieto et al	2017	Ringer’s solution (400)	Aerobic for 5 days and anaerobic for 7 days	Yes	Yes	NA	IDSA	0.71	0.96	27	2	11	48	88
Fernandez et al	2017	Ringer’s solution (400)	Aerobic for 5 days and anaerobic for 7 days	Yes	Yes	20 CFU/plate	NNIS	0.85	0.99	110	2	20	366	498
Fernandez et al	2015	Ringer’s solution (400)	Aerobic for 5 days and anaerobic for 7 days	Yes	Yes	20 CFU/plate	IOF, H, M	0.88	1.00	21	0	3	174	198
Bogut et al	2014	Ringer’s solution (400)	Aerobic and anaerobic for 14 days	Yes	Yes	NA	IOF, H	0.75	0.97	17	2	5	52	76
Ryu et al	2014	Ringer’s solution (400)	Aerobic for 2–5 days and anaerobic for 7–14 days	Yes	Yes	20 CFU/plate	IOF, H, M	0.77	1.00	23	0	7	15	45
Dapunt et al	2014	Ringer’s solution (400)	Aerobic and anaerobic for a maximum of 14 days	Yes	No	NA	NA	0.92	0.67	23	17	2	35	77
Portillo et al	2014	Thioglycolate broth (50–200)	Aerobic for 7 days and anaerobic for 14 days	No	Yes	50 CFU/ml	IOF, H, M	0.81	0.99	56	1	13	161	231
Drago et al	2013	Sterile saline	Aerobic for 24 h and anaerobic for 48 h	Yes	Yes	5 CFU/plate	IOF, H, M	0.71	0.94	30	2	12	32	76
Cazanave et al	2013	Ringer’s solution (400)	Aerobic for 2–4 days and anaerobic for 14 days	Yes	Yes	20 CFU/plate	NA	0.73	0.98	105	5	39	285	434
Janz et al	2013	Ringer’s solution	Aerobic and anaerobic for 14 days	No	Yes	NA	IOF, H	0.89	0.72	33	18	4	47	102
Janz et al	2013	Ringer’s solution	Aerobic and anaerobic for 14 days	No	Yes	5 CFU/plate	IOF, H, M	0.91	0.81	21	7	2	29	59
Portillo et al	2012	Thioglycolate broth (50–200)	Aerobic for 7 days and anaerobic for 14 days	No	Yes	50 CFU/ml	NA	0.6	0.99	21	1	14	99	135
Gomez et al	2012	Ringer’s solution (400)	Aerobic for 4 days and anaerobic for 14 days	Yes	Yes	2 CFU/ml	IOF, H	0.73	0.98	99	5	36	226	366
Portillo et al	2012	Thioglycolate broth (50–200)	Aerobic for 7 days and anaerobic for 14 days	No	Yes	50 CFU/ml	IOF, H, M	0.71	1.00	17	0	7	62	86
Vergidis et al	2011	Ringer’s solution (400)	Aerobic for 2–4 days and anaerobic for 14 days	Yes	Yes	20 CFU/plate	IOF, H, M	0.89	1.00	8	0	1	27	36
Piper et al	2009	Ringer’s solution (400)	Aerobic for 5 days and anaerobic for 7 days	Yes	Yes	20 CFU/plate	IOF, H	0.67	0.98	22	2	11	99	134
Trampuz et al	2007	Ringer’s solution (400)	Aerobic for 5 days and anaerobic for 7 days	Yes	Yes	5 CFU//plate	IOF, H	0.79	0.99	62	3	17	249	331
Trampuz et al	2006	Ringer’s solution (100)	Aerobic for 5 days and anaerobic for 7 days	No	No	NA	IOF, H	0.75	0.87	18	7	6	47	78

*NA* not available; *CFU* colony-forming units; *MSIS* Musculoskeletal Infection Society; *ICM* International Consensus Meeting; *IDSA* Infectious Diseases Society of America; *EBJIS* European Bone and Joint Infection Society; *NNIS* National Nosocomial Infections Surveillance; *H* histological examination; *IOF* intraoperative finding; *M* microbiological or laboratory examination; *Sen* Sensitivity; *Spe* Specificity; *TP* true positive; *FP* false positive; *FN* false negative; *TN* true negative

### Heterogeneity analysis

The data extracted in this study were imported into Meta DiSc software for threshold effects analysis, and the Spearman correlation coefficient between logarithms of sensitivity and logarithms of (1-specificity) was 0.249 (*p* = 0.132 > 0.05), indicating that there was no threshold effect in this study. Meanwhile, by drawing the asymmetric SROC curve, there was no “shoulder arm shape”, which further showed that heterogeneity might be independent of the threshold effect in our study (Fig. 4b). Furthermore, the Cochran-Q test of DOR showed that Cochran-Q was 107.71 (*p* < 0.001), indicating heterogeneity caused by a nonthreshold effect.

### Threshold and diagnostic accuracy of SFC for PJI

For the cutoff value, 10 and 6 studies used common thresholds of 50 CFU/ml and 20 CFU/plate, respectively, while the remaining 22 studies used different thresholds.

**Fig. 3:**
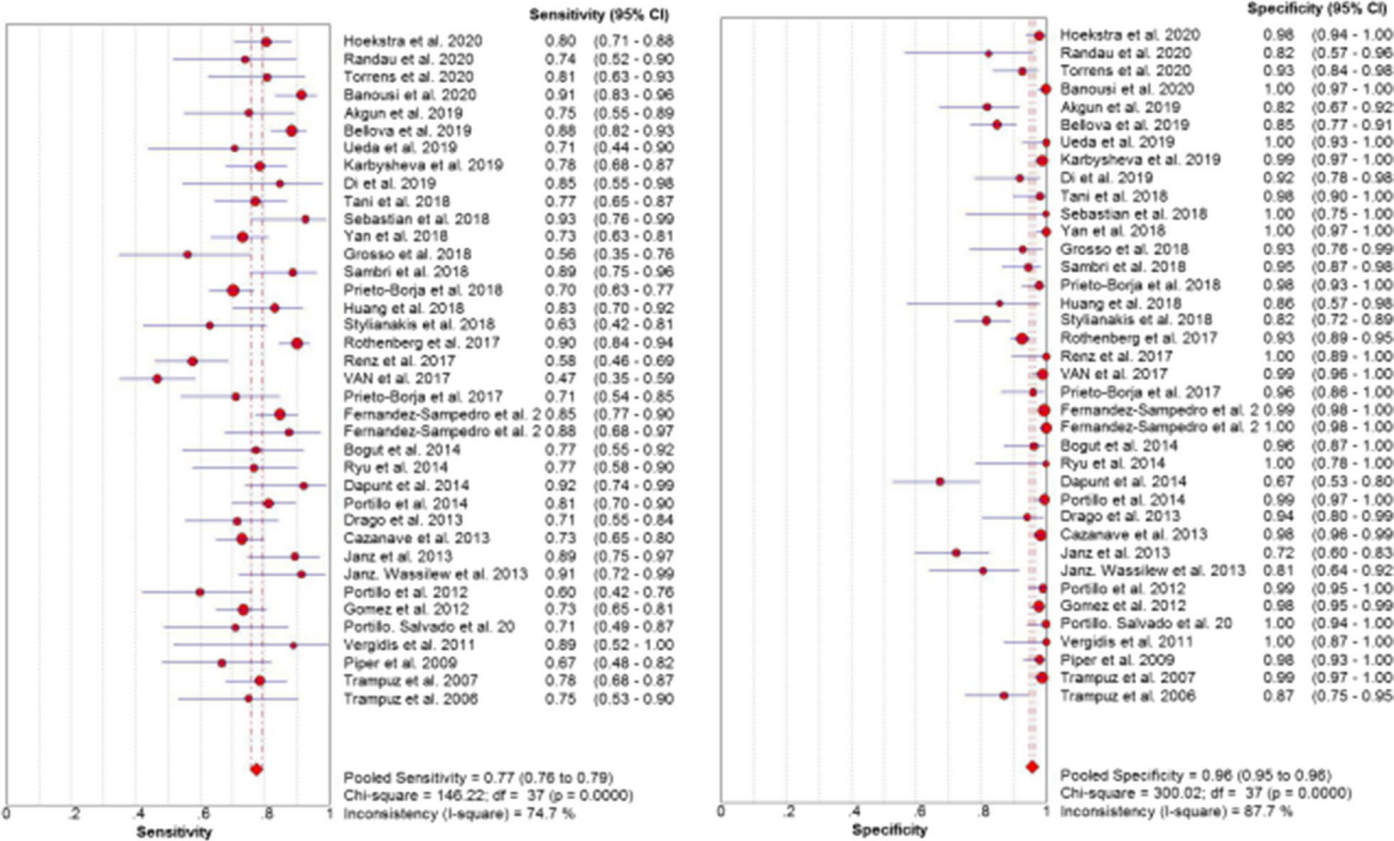
Forest plots with sensitivity and specificity of sonication fluid cultures for PJI

**Fig. 4:**
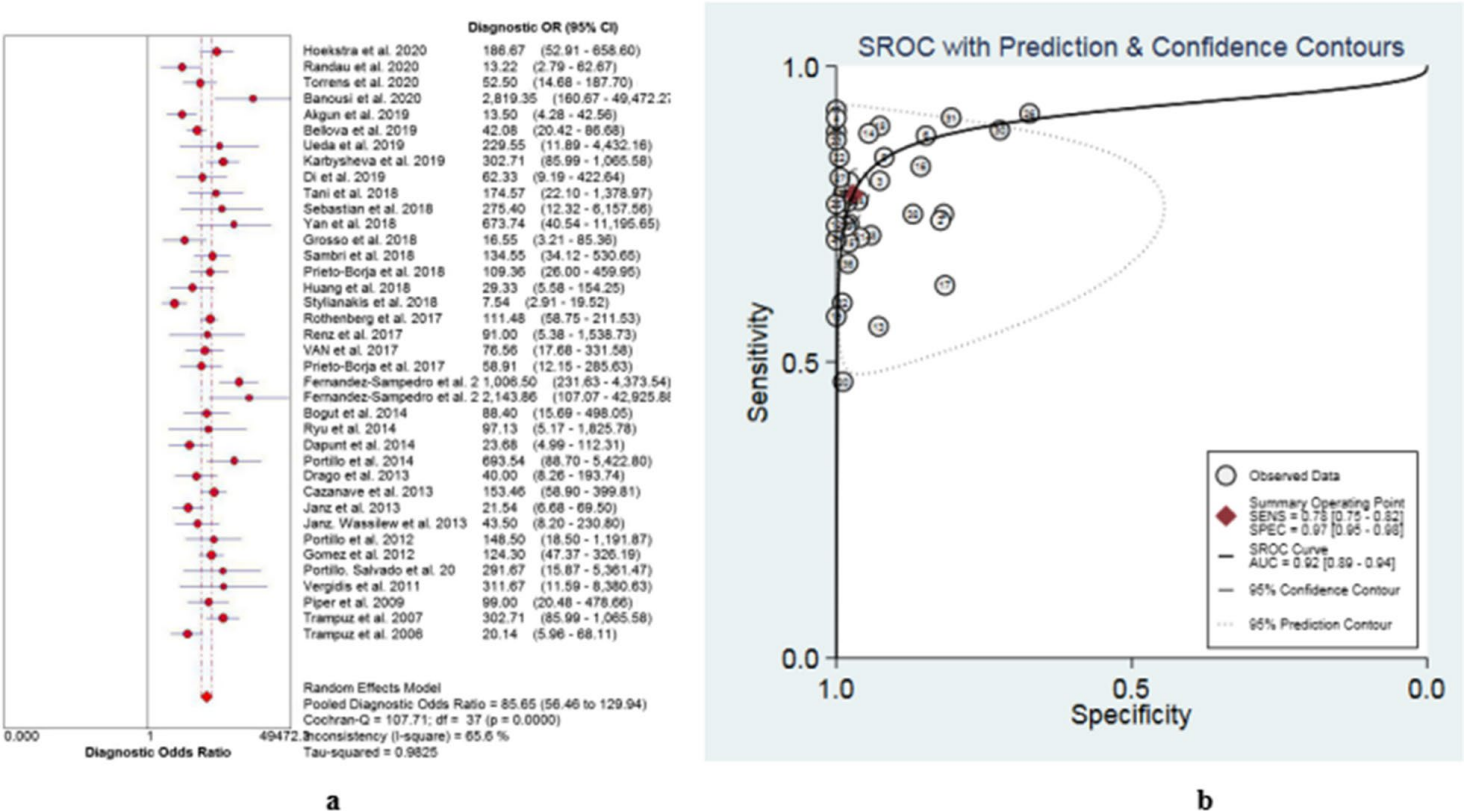
Pooled diagnostic odds ratio (**a**) and SROC curve of included studies (**b**)

**Fig. 5:**
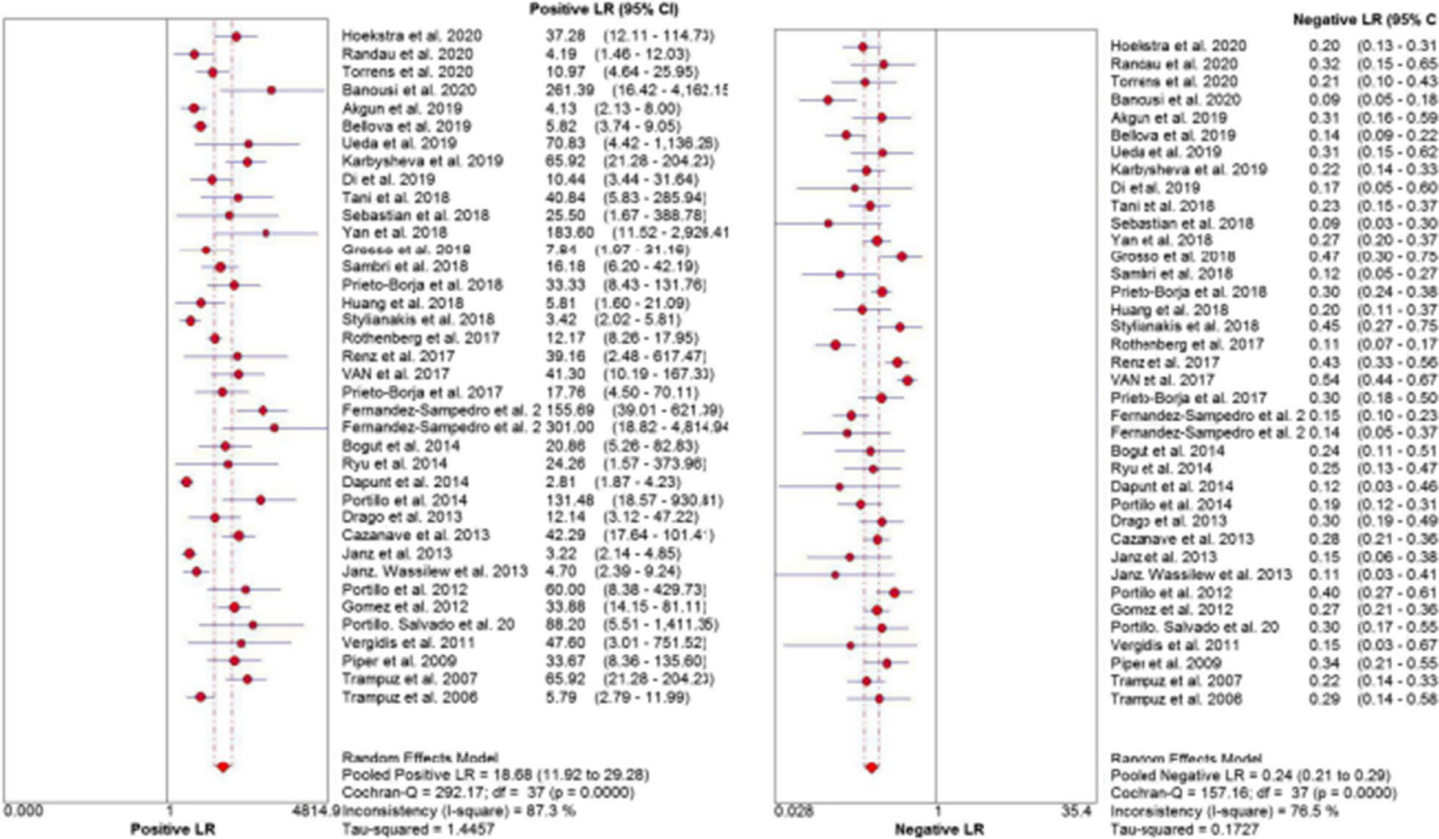
Forest plots of pooled likelihood ratio (positive likelihood ratio and negative likelihood ratio)

Significant heterogeneity was found in sensitivity (*I*^2^ = 74.7%, *p* < 0.01), specificity (*I*^2^ = 87.7%, *p* < 0.01), PLR (*I*^2^ = 87.3%, *p* < 0.01), NLR (*I*^2^ = 76.5%, *p* < 0.01) and DOR (*I*^2^ = 65.6%, *p* < 0.01) (Fig. 3, Fig. 4a, Fig. 5); thus, the random-effects model was performed. As shown in Table 3, the pooled sensitivity and specificity of SFC for diagnosing PJI were 0.77 (95% CI, 0.76–0.79) and 0.96 (95% CI, 0.95–0.96), respectively. The pooled DOR was 85.65 (95% CI, 56.46–129.94) (Fig. 4a). The AUC of SFC for PJI was 0.92 (95% CI, 0.89–0.94) (Fig. 4b).

**Table 3:** Subgroup analysis of sonication fluid cultures for PJI diagnosis

Subgroup analyses	No. of studies	No. of joints	Estimates (95% CI)					
			Sensitivity	Specificity	PLR	NLR	AUC	DOR
Overall studies	38	6302	0.77 (0.76–0.79)	0.96 (0.95–0.96)	18.68 (11.92–29.28)	0.24 (0.21–0.29)	0.92 (0.89–0.94)	85.65 (56.46–129.94)
*Study design*								
Prospective	19	3081	0.78 (0.76–0.81)	0.97 (0.96–0.98)	28.46 (11.99–67.57)	0.23 (0.18–0.29)	0.91 (0.89–0.93)	134.96 (58.53–311.20)
Retrospective	17	3068	0.76 (0.74–0.79)	0.95 (0.94–0.96)	15.57 (9.85–24.61)	0.25 (0.20–0.32)	0.94 (0.91–0.95)	71.85 (47.96–107.65)
*Sample site(s)*								
H, K, S, E or A	7	1093	0.74 (0.70–0.78)	1.00 (0.99–1.00)	114.20 (43.84–297.47)	0.25 (0.21–0.29)	0.99 (0.99–1.00)	386.56 (18.6–1077.46)
H, K, S	4	698	0.78 (0.74–0.82)	0.88 (0.84–0.91)	7.54 (5.40–1.54)	0.23 (0.19–0.28)	0.92 (0.90–0.94)	52.23 (29.86–91.34)
H, K	18	3818	0.79 (0.76–0.81)	0.97 (0.96–0.97)	19.32 (10.71–34.87)	0.22 (0.17–0.29)	0.93 (0.90–0.95)	98.18 (52.62–183.16)
H or K or S	8	617	0.76 (0.70–0.81)	0.91 (0.88–0.93)	7.41 (5.46–10.05)	0.27 (0.21–0.34)	0.87 (0.84–0.90)	34.76 (20.72–58.32)
*Publication year*								
≥ 2014	27	4465	0.78 (0.76–0.80)	0.96 (0.95–0.96)	18.55 (10.88–31.62)	0.23 (0.18–0.29)	0.92 (0.90–0.94)	89.08 (51.92–152.86)
< 2014	11	1837	0.75 (0.71–0.78)	0.96 (0.95–0.97)	19.50 (7.70–49.43)	0.28(0.24–0.32)	0.87(0.84–0.90)	81.62 (43.84–151.95)
*Cutoff value*								
50 CFU/ml	10	1651	0.76 (0.72–0.79)	0.96 (0.95–0.97)	21.44 (9.90–46.42)	0.26 (0.17–0.40)	0.94 (0.92–0.96)	91.26 (42.43–196.31)
20 CFU/plate	6	1345	0.78 (0.74–0.82)	0.99 (0.98–1.00)	62.89 (34.98–113.07)	0.22 (0.18–0.27)	0.97 (0.95–0.98)	247.74 (129.61–473.53)
20 CFU/ml	2	93	0.75 (0.61–0.86)	0.95 (0.83–0.99)	9.97 (2.91–34.16)	0.23 (0.04–1.26)	0.50 (0.47–0.55)	49.03 (3.31–725.31)
5 CFU/plate	3	466	0.78 (0.71–0.85)	0.96 (0.94–0.98)	15.09 (2.41–94.53)	0.24 (0.16–0.35)	0.94 (0.91–0.96)	87.74 (21.74–354.16)
2 CFU/ml	2	595	0.73 (0.67–0.79)	0.99 (0.97–1.00)	50.31 (21.24–119.15)	0.27 (0.22–0.34)	0.50 (0.49–0.52)	185.54 (74.48–462.16)
*Number (joints)*								
≥ 100	21	5193	0.77 (0.75–0.79)	0.97 (0.96–0.97)	31.99 (16.64–61.49)	0.23 (0.19–0.29)	0.94 (0.91–0.95)	139.61 (79.32–245.72)
< 100	17	1109	0.78 (0.74–0.81)	0.91 (0.88–0.93)	7.93 (6.18–10.17)	0.25 (0.21–0.30)	0.87 (0.84–0.90)	37.86 (25.60–55.98)
*Antibiotic use before revision*								
Yes	19	3799	0.79 (0.76–0.81)	0.97 (0.96–0.98)	31.24 (17.62–55.40)	0.23 (0.19–0.28)	0.93 (0.91–0.95)	132.06 (81.46–214.07)
No	13	1825	0.74 (0.71–0.77)	0.94 (0.92–0.95)	11.07 (5.28–23.24)	0.27 (0.19–0.38)	0.91 (0.88–0.93)	48.79 (21.16–112.50)
*Sample processing time (after prosthesis removal)*								
Within 6 h	21	2794	0.75 (0.73–0.78)	0.95 (0.94–0.96)	15.09 (8.29–27.48)	0.25 (0.20–0.32)	0.92 (0.90–0.94)	66.01 (36.77–118.51)
Within 24–48 h	3	431	0.70 (0.64–0.76)	0.98 (0.95–0.99)	29.62 (11.44–76.71)	0.30 (0.25–0.37)	0.80 (0.78–0.83)	97.88 (35.62–268.93)
*Patient enrollment*								
Consecutive	34	5742	0.77 (0.75–0.79)	0.96 (0.95–0.97)	19.16 (12.11–30.33)	0.24 (0.20–0.29)	0.92 (0.89–0.94)	87.67 (55.86–137.60)
NA (Not provided)	4	560	0.79 (0.72–0.85)	0.94 (0.91–0.96)	11.14 (7.66–16.20)	0.22 (0.17–0.30)	0.87 (0.84–0.90)	78.00 (36.93–164.74)
*Solution for prosthesis*								
Ringer’s solution	23	4342	0.79 (0.77–0.81)	0.95 (0.94–0.96)	16.79 (9.57–29.48)	0.23 (0.18–0.29)	0.92 (0.90–0.94)	81.45 (47.75–138.95)
sterile saline	8	622	0.74 (0.68–0.79)	0.93 (0.90–0.96)	9.94 (5.17–19.10)	0.26 (0.18–0.38)	0.92 (0.89–0.94)	45.82 (20.80–100.93)
Thioglycolate broth	3	452	0.73 (0.65–0.81)	0.99 (0.98–1.00)	96.22 (27.43–337.50)	0.27 (0.20–0.36)	0.99 (0.99–1.00)	330.02 (91.33–1192.51)
*Centrifugation*								
Yes	24	4440	0.78 (0.76–0.80)	0.96 (0.95–0.97)	20.64 (11.67–36.49)	0.23 (0.18–0.28)	0.93 (0.90–0.95)	95.53 (56.83–160.59)
No	11	1252	0.74 (0.70–0.78)	0.93 (0.91–0.95)	12.21 (5.68–26.25)	0.28 (0.21–0.37)	0.89 (0.86–0.91)	53.34 (25.25–112.72)
*Vortexing*								
Yes	32	5261	0.78 (0.76–0.80)	0.96 (0.95–0.97)	19.08 (11.96–30.46)	0.24 (0.20–0.29)	0.92 (0.90–0.94)	87.14 (55.07–137.87)
No	3	431	0.73 (0.67–0.79)	0.87 (0.82–0.91)	7.48 (1.40–40.02)	0.29 (0.22–0.38)	0.91 (0.89–0.92)	36.54 (11.83–112.90)
*Culture period*								
Aerobic and anaerobic (≦7d)	10	2045	0.73 (0.69–0.76)	0.98 (0.97–0.99)	33.41 (14.41–77.47)	0.27 (0.20–0.37)	0.93 (0.91–0.95)	125.03 (54.46–287.03)
Aerobic (≦7d) and anaerobic (7-14d)	15	2732	0.81 (0.79–0.84)	0.96 (0.95–0.97)	21.01 (10.93–40.39)	0.21 (0.16–0.27)	0.93 (0.91–0.95)	107.69 (54.11–214.32)
Aerobic and anaerobic (7-14d)	10	915	0.75 (0.71–0.80)	0.88 (0.85–0.91)	6.81 (3.80–12.20)	0.26 (0.18–0.37)	0.90 (0.87–0.92)	34.90 (19.12–63.70)
*Reference standard*								
MSIS or ICM	13		0.81 (0.78–0.84)	0.94 (0.93–0.96)	12.41 (7.67–20.06)	0.21 (0.14–0.31)	0.94 (0.92–0.96)	68.58 (39.56–118.89)
IDSA or EBJIS	6		0.73 (0.69–0.77)	0.99 (0.98–1.00)	38.49 (17.73–83.54)	0.26 (0.19–0.37)	0.80 (0.77–0.83)	170.30 (61.47–471.82)
Other	19		0.77 (0.74–0.79)	0.95 (0.95–0.97)	20.17 (9.37–43.42)	0.26 (0.22–0.31)	0.87 (0.84–0.90)	87.12 (43.37–175.01)
*Geographical location*								
USA	9		0.78 (0.74–0.81)	0.96 (0.95–0.97)	20.96 (11.04–39.78)	0.25 (0.19–0.34)	0.94 (0.92–0.96)	93.09 (51.04–169.78)
Europe	25		0.77 (0.75–0.79)	0.95 (0.94–0.96)	17.36 (9.74–30.94)	0.24 (0.19–0.30)	0.92 (0.89–0.94)	82.68 (46.95–145.59)
Asian	4		0.78 (0.72–0.84)	0.99 (0.96–1.00)	29.65 (4.37–201.33)	0.24 (0.17–0.34)	0.92 (0.89–0.94)	140.81 (25.88–766.17)

*NA* not available; *H* Hip; *K* Knee; *S* Shoulder; *E* Elbow; *A* Ankle; *PLR* positive likelihood ratio; *NLR* negative likelihood ratio; *AUC* area under the curve; *CI* confidence interval; *DOR* diagnostic odds ratio; *CFU* colony-forming units; *MSIS* Musculoskeletal Infection Society; *ICM* International Consensus Meeting; *IDSA* Infectious Diseases Society of America; *EBJIS* European Bone and Joint Infection Society; d days

### Evaluation of the clinical utility

**Fig. 6:**
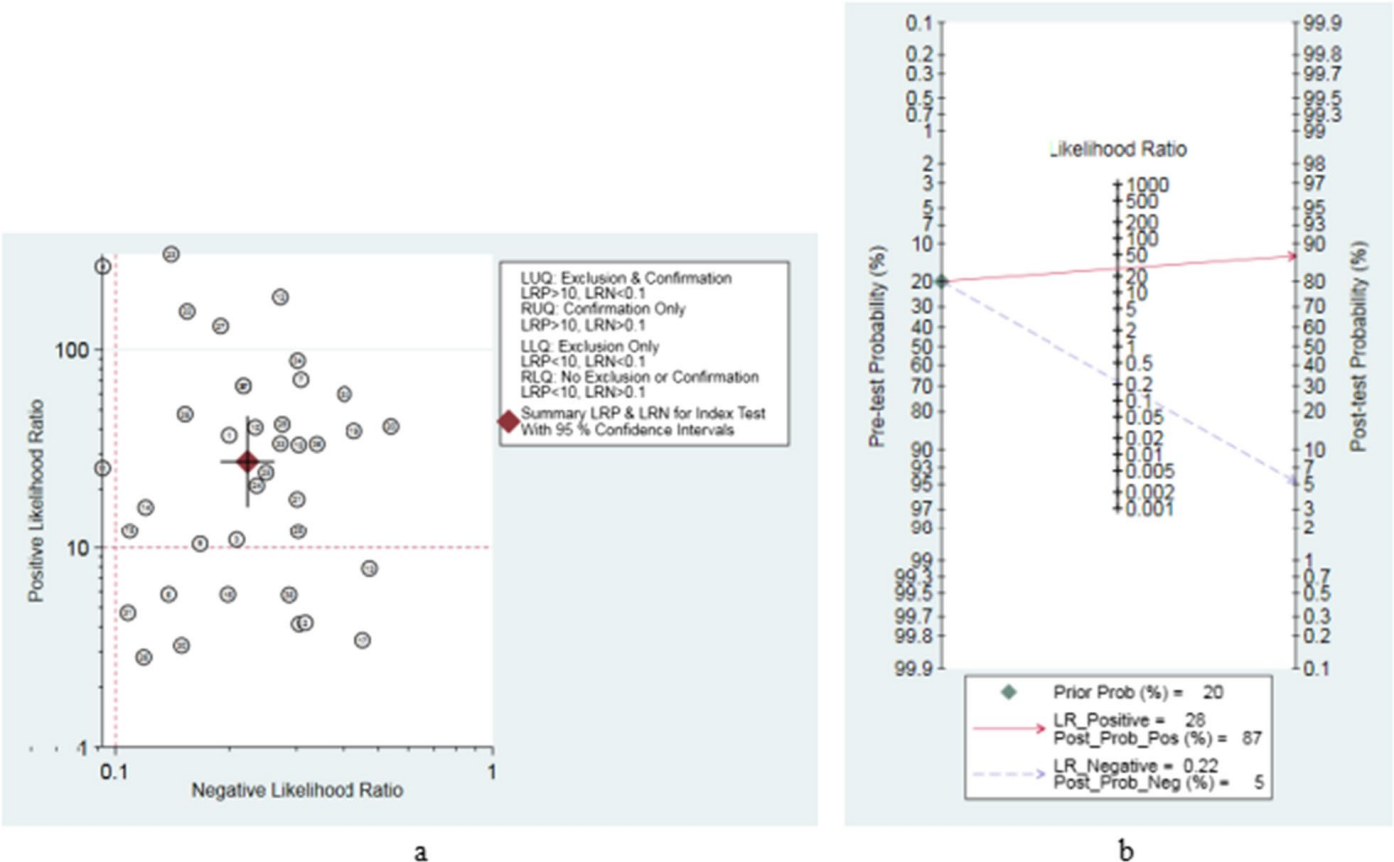
Pooled likelihood ratio scatter diagrams (**a**) and Fagan’s nomogram of sonication fluid cultures for diagnosis of PJI (**b**)

As shown in Table 3 and Fig. 6a, the pooled PLR and NLR of SFC for PJI diagnosis were 18.68 (95% CI, 11.92–29.28) and 0.24 (95% CI, 0.21–0.29), respectively. According to previous studies, the incidence of PJI is approximately 20% in revision arthroplasty. Therefore, a 0.2 pretest probability was used to calculate the posttest probability by the likelihood ratio and pretest probability [55]. The posttest probability of PJI was 5%, indicating negative SFC results (Fig. 6b).

### Subgroup analysis

The Cochran-Q test of DOR showed that the heterogeneity was caused by a nonthreshold effect, and the heterogeneity of this study was large. Therefore, we performed the following subgroup analysis to explore the source of heterogeneity according to the study design, the sample type, the publication year, the threshold used in the study, the number of joints, reference standard, antibiotic use before revision, sample processing time, solution for prosthesis, centrifugation, vortexing, culture period, and geographical location. The subgroup analysis showing the pooled sensitivity, specificity, PLR, NLR, DOR, and AUC of each subgroup are presented in Table 3. The pooled sensitivity, specificity, PLR, NLR, DOR, and AUC estimates for detecting PJI by using SFC at 50 CFU/ml were 0.76 (95% CI, 0.72–0.79), 0.96 (95% CI, 0.95–0.97), 21.44 (95% CI, 9.90–46.42), 0.26 (95% CI, 0.17–0.40), 91.26 (95% CI, 42.43–196.31), and 0.94 (95% CI, 0.92–0.96), respectively. In the subgroup of the reference standard, the pooled sensitivity, specificity, and AUC were 0.81 (95% CI, 0.78–0.84), 0.94 (95% CI, 0.93–0.96), and 0.94 (95% CI, 0.92–0.96), respectively, for MSIS or ICM, while they were 0.73 (95% CI, 0.69–0.77), 0.99 (95% CI, 0.98–1.00), and 0.80 (95% CI, 0.77–0.83), respectively, for IDSA or EBJIS.

### Sensitivity analysis and publication biases

**Fig. 7:**
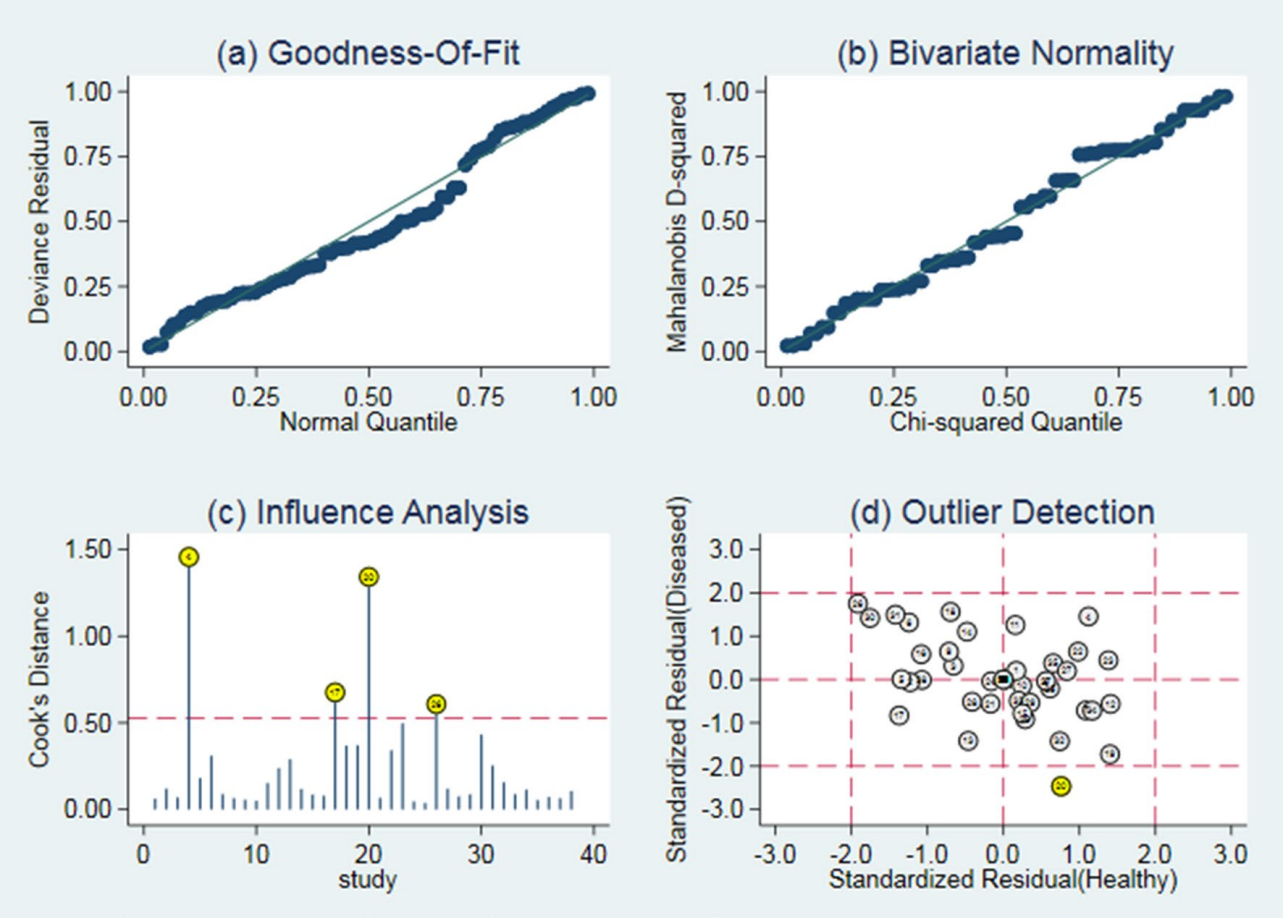
Sensitivity analysis: **a** Goodness of fit, **b** bivariate normality, **c** influence analysis, **d** outlier detection

**Fig. 8:**
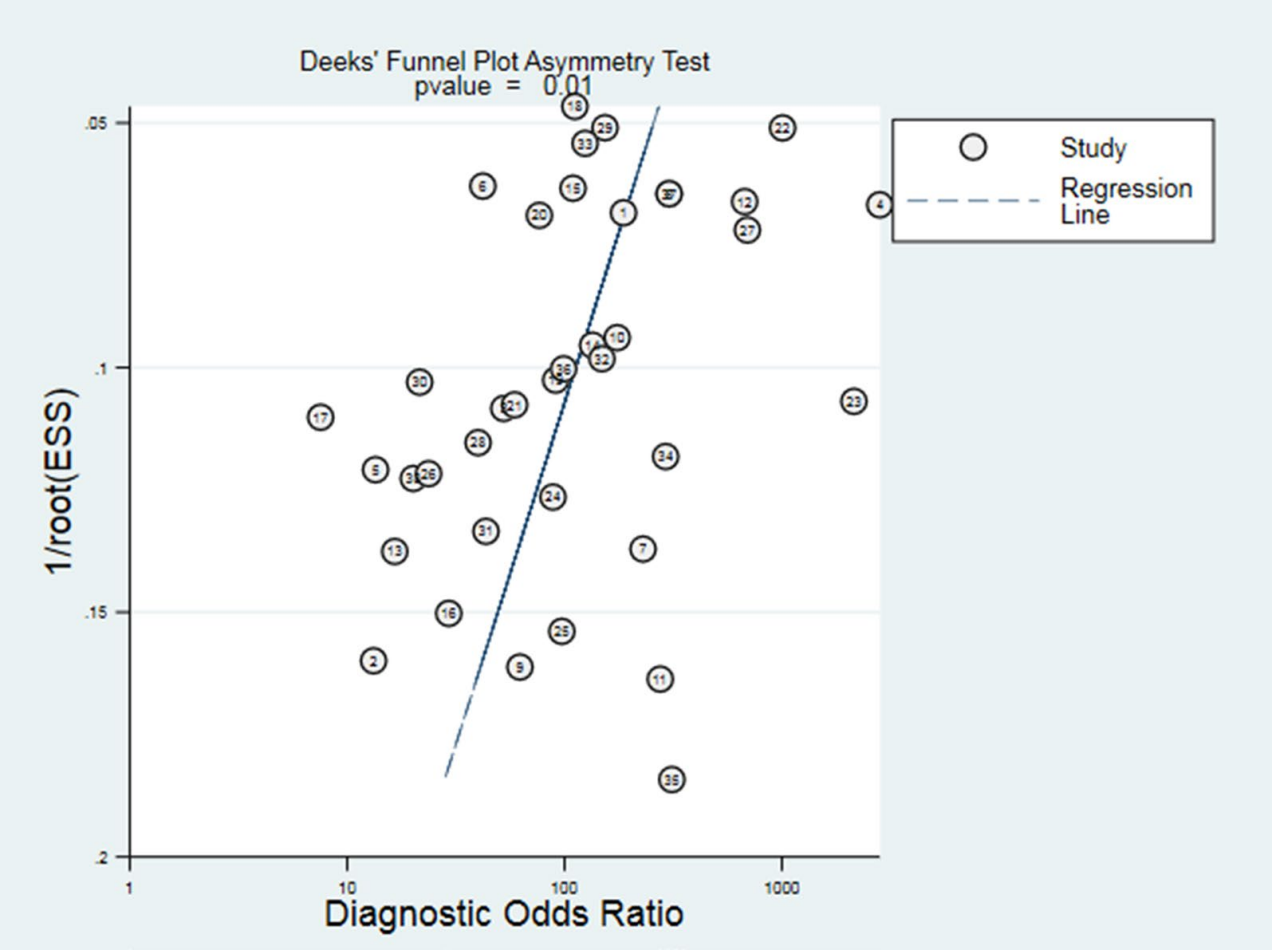
Funnel plot for publication bias assessment of included studies

As shown in Fig. 7, there were four original studies with relatively strong sensitivity, and other studies did not cause sensitivity in the pooled results. Overall, the results of our study should be relatively sound. The funnel plot based on Deeks’ test of the pooled DOR was asymmetric, indicating a possible publication bias (*p* = 0.01 < 0.05) (Fig. 8).

## Discussion

As the diagnosis of PJI remains a challenge and PJI brings a huge economic burden to society, an increasing number of orthopaedists pay attention to this devastating complication after TJA. Accurate diagnosis is the key to treating PJI because it has a major influence on the direction of treatment (antibiotic use and surgical rehabilitation) and the course of intervention. Therefore, an increasing number of preoperative and intraoperative tests have been performed to diagnose PJI [7–9, 13, 27, 28, 32–34, 40]. Historically, intraoperative tissue cultures have been used as the gold standard. It is known that bacteria can exist in two main forms, planktonic and sessile. Banousi et al. [24] suggested that the biofilms formed by bacteria at the surface of implants are not only a major factor for chronic PJI but also one of the main causes for the lack of positive cultures in periprosthetic soft tissue samples obtained intraoperatively. Moreover, bacteria in the form of biofilms are resistant to antibiotics and difficult to detect by conventional tissue culture. Therefore, previous studies reported that intraoperative tissue cultures lacked adequate sensitivity (ranging from 0.51 to 0.90) and specificity (ranging from 0.67 to 1.00) [15, 20, 30, 34, 38, 56]. Moreover, Renz et al. [38] found that the sensitivity of intraoperative tissue cultures for the diagnosis of PJI was only 51.0%. Parvizi et al. [56] suggested that there was no significant difference in clinical features between chronic deep PJI and aseptic joint loosening. Fortunately, with the development of medicine, various detection methods have been found for the diagnosis of PJI, such as laboratory tests (e.g., white blood cell count, erythrocyte sedimentation rate, C-reactive protein, interleukin-6, alpha-defensin, procalcitonin, D-dimer, and fibrinogen), synovial fluid characteristics, histopathological studies, microbiological studies (e.g., conventional synovial fluid and tissue cultures, and SFC), and radiological studies. To the best of our knowledge, recovering qualified bacteria from samples is a prerequisite for improving diagnostic accuracy. As Janz et al. [47] showed, the true diagnostic ability of cultures depends on the accurate recovery of bacteria from samples. Fortunately, Tunney et al. [57] suggested that sonication can dislodge adherent bacteria from explanted prosthetic surfaces by ultrasound. In addition, a study by Trampuz et al. [13] confirmed that cultures of microorganisms from removed orthopaedic implants are more sensitive than tissue cultures. To date, many studies have reported that SFC improves the accuracy of PJI diagnosis [13, 15, 16, 18, 30, 35, 45, 48, 52]. Meanwhile, we found that SFC had a high sensitivity of 0.77 (95% CI, 0.76–0.79) and a very high specificity of 0.96 (95% CI, 0.95–0.96) for the diagnosis of PJI.

As an important diagnostic method for PJI, SFC is still widely used in clinical practice. Since this technique is simple, it can be employed in most microbiology laboratories. Trampuz et al.[13] proposed that another advantage of SFC is the improved detection rate of polymicrobial PJI; meanwhile, they also popularized the application of sonication to detect PJI and described a significant improvement in the sensitivity of SFCs (78.5%) when compared with periprosthetic tissue cultures (PTCs) (60.8%). Since then, SFCs have shown superior sensitivity as opposed to tissue cultures in the diagnosis of PJI, which has been reported in many previous studies [13, 15–18, 24, 30–32, 35, 44–47, 51, 52]. For example, Banousi et al.[24] reported on 234 patients with revision arthroplasty (91 with PJI), and SFCs were more sensitive than PTCs (91% vs. 43%), while specificities were similar (100% vs. 100%). In another study of 200 patients and 276 samples, the culture sensitivities of sonicate fluid, periprosthetic tissue, synovial fluid and a combination of periprosthetic tissue and/or synovial fluid were 69.5, 52.8, 54.8 and 60.2%, respectively; the specificities were 97.6, 90.3, 93.0 and 89.9%, respectively [35]. In a study of 503 patients who underwent revision total hip arthroplasty and total knee arthroplasty, Adam et al. [15] demonstrated that the sensitivity of SFC (0.97; 95% CI, 0.89–0.99) was greater than that of synovial fluid culture (0.57; 95% CI, 0.44–0.69) and PTC (0.70; 95% CI, 0.58–0.80), although the specificity of SFC was not significantly different from that of synovial culture or tissue culture (0.90, 95% CI 0.72–0.97 vs. 1.0, 95% CI 0.86–1.0; 0.90, 95% CI 0.72–0.97 vs. 0.97, 95% CI 0.81–1.0, respectively). Sonication of retrieved implants in particular seems to have additional value in the detection of low-virulent biofilm-producing microorganisms that cause chronic PJIs. Trampuz et al. [13] reported that the sensitivities of PTCs and SFCs were 60.8% and 78.5%, respectively, and found that SFCs detected 14 cases but not PTCs. One recent study claimed that SFC detected positive results in 8 of 87 patients with PJI, while PTC was negative, and Hoekstra et al. emphasized that SFC should be used to rule out infection in patients with suspected early aseptic loosening of the prosthesis and negative preoperative synovial fluid culture [23]. Furthermore, previous studies have found a strong trend toward greater sensitivity of SFC against PTC in chronic/delayed PJI [29, 31, 35, 58]. In addition, Prieto et al. [35] further revealed that SFC (91.3%) was more sensitive for all infection types of implants than PTC (60.0%), synovial fluid (63.2%) and a combination of PTC and/or synovial fluid (66.7%); when conventional cultures were combined with SFC, the sensitivity improved significantly in total (from 60.2 to 77.1%) and delayed PJI (from 45.1 to 71.7%). It is interesting to note that SFC significantly improves pathogen detection, especially for patients who were administered antimicrobial therapy [13, 26, 32], and the sensitivity of SFCs was significantly superior to that of PTCs in patients who received antibacterial treatment within 14 days before operation (75.0% vs. 45.0%, p < 0.001), as reported by Trampuz et al. [13].

Various factors, including antibiotic use and specimen contamination, may affect the accuracy of SFC diagnosis [45, 47, 49, 52]. Meanwhile, in our subgroup analysis, we found that the diagnostic ability of SFC varied with the study design, cutoff value (CFU value), reference standard, sample type (such as hip, knee, shoulder and elbow), number of joints, antibiotic use before revision, sample processing time, solution used for prosthesis, centrifugation, vortexing, culture period, geographic location and year of publication (Table 3). It is well known that traditional methods such as PTCs also have the same disadvantages for PJI in patients with these conditions.

Since no consensus has been reached on the use of a single threshold to date, different cutoff values of SFC (0.1–50 CFU/mL) were applied in recent studies to diagnose PJI [13–17, 20–52]. For example, due to different infection criteria, the culture protocols and reference standards in the study of Piper et al. and Grosso et al. differed [16, 37]. Using a cutoff of 20 CFU/mL, Piper et al. [16] reported an increased SFC sensitivity compared with PTC (66.7% vs. 54.5%), and specificities were not significantly different (98% vs. 95.1%), whereas a higher sensitivity (96%) and lower specificity (75%) for standard intraoperative cultures as well as a lower sensitivity for SFCs (56%) were reported by Grosso et al. [37]. Our results are similar to those of Piper et al., and we found a higher sensitivity (78%), and specificity (99%) for SFCs in this meta-analysis at the 20 CFU/plate cutoff. In our study, the superior sensitivity and specificity of SFC for detecting hip and knee prosthesis infections were 0.78 (95% CI, 0.76–0.81), 0.97 (95% CI, 0.96–0.97), respectively. In fact, previous studies have concluded that SFCs as a successful diagnostic tool may be most beneficial for detecting organisms in this population of patients [13, 58, 59]. Because the PJI diagnostic threshold of SFC was different for various joints, the sensitivity and specificity of diagnosis were also different. Therefore, the appropriate cutoff value of SFC in the diagnosis of PJI still needs to be studied.

In addition, the findings of comparable studies confirmed that previous administration of antibiotics had no effect on the sensitivity of SFC [60, 61]. However, our subgroup analysis showed a trend toward higher sensitivity and specificity of SFC in the antibiotic groups as opposed to when antibiotics were not administered (79% vs. 74%, 97% vs. 94%, respectively). In this meta-analysis, it must be noted that the antibiotic group was characterized by a relatively large sample size (n = 3799). Furthermore, the criteria used for the definition of PJI differed between studies, including MSIS, ICM, IDSA, EBJIS, and so on. In our study, higher sensitivity was shown when using MSIS or ICM as a reference standard (0.81; 95% CI, 0.78–0.84). To the best of our knowledge, the duration of the incubation period remains controversial. In our study, two- to 14-day incubations were reported [13–17, 20–52]. For PJI caused by low-virulence microorganisms, however, most studies suggested prolonging the anaerobic culture period for up to 14 days [14, 15, 17, 21, 22, 24, 25, 29, 32–34, 36, 38, 42–47, 49–51]. By comparing the time to culture tissue and sonication fluid in PJI and aseptic failure, Butler-Wu SM et al. [62] found that anaerobic organisms were detected in SFC up to 13 days, whereas aerobes required only 7 days of incubation. In this meta-analysis, 10 studies detected aerobic and anaerobic bacteria in SFC within 7 days, 15 studies detected aerobic bacteria within 7 days and anaerobic bacteria within 7–14 days, and aerobic and anaerobic bacteria in SFC were detected in 10 studies within 7–14 days. In addition, the subgroup analysis showed that patients with the second condition (aerobic (≤ 7 days) and anaerobic (7–14 days) culture were performed in SFC) had the highest sensitivity of 0.81 (95% CI, 0.79–0.84). Therefore, our results supported that 7-day aerobic culture and 7–14-day anaerobic culture may improve the sensitivity of PJI diagnosis. Of note, previous studies [13, 17, 58, 59, 63] have reported that SFC has gained acceptance in detecting hip and knee prosthetic infections. Nevertheless, Carlos et al. [21] did not support the routine use of SFC to detect infection in shoulder implants. Consistent with previous studies, in our study, SFC had the best sensitivity and specificity for detecting hip and knee prosthesis infection, with 0.79 (95% CI, 0.76–0.81) and 0.97 (95% CI, 0.96–0.97), respectively. Additionally, in this meta-analysis, 34 studies recorded the implants that were covered with solution. It should be mentioned that Ringer's solution was the most commonly used solution (23/38, 60.5%), with a relatively high sensitivity and specificity of 0.79 (95% CI, 0.76–0.81), and 0.95 (95% CI, 0.96–0.97), respectively. Portillo et al. [45] reported that the vortexing-sonication procedure demonstrated higher biofilm removal efficiency than vortexing alone. In this study, compared to SFC without the use of centrifugation or vortexing, SFC with the use of centrifugation or vortexing had a higher sensitivity (centrifugation, 0.78 [CI, 0.76–0.80] versus 0.74 [CI, 0.70–0.78] and vortexing, 0.78 [CI, 0.76–0.80] versus 0.73 [CI, 0.67–0.79], respectively) and specificity (centrifugation, 0.96 [CI, 0.95–0.97] versus 0.93 [CI, 0.91–0.95] and vortexing, 0.96 [CI, 0.95–0.97] versus 0.87 [CI, 0.82–0.91], respectively).

Although an excellent meta-analysis on SFC in the diagnosis of PJI has been published by Zhai et al. [64], we noted that their four articles were inconsistent with other studies and should not be included in the meta-analysis. One of their studies involved 16S rRNA gene PCR analysis for the diagnosis of PJI [65]. One study involved multiplex PCR of sonication fluid for the diagnosis of PJI [66]. The subjects of the other two studies were not only considering joint prostheses but also other orthopaedic implants, such as fixation devices and spinal devices[58, 67]. Therefore, the reliability of their conclusions may be limited. Compared with Zhai et al. [64], our meta-analysis included more up-to-date studies and patients (6302 patients in 38 studies) after rigorous screening and literature quality evaluations. In addition, our pooled AUC of SFC for the diagnosis of PJI was 0.92 (95% CI, 0.89–0.94), which was higher than the results of Zhai et al. (AUC = 0.89). Furthermore, the overall pooled sensitivity and specificity were calculated to be 0.77 and 0.96, respectively. However, heterogeneity still exists in our study. For example, in the 4 studies from 2020, inconsistent results are shown in "Forest plots with sensitivity and specificity of sonication fluid cultures for PJI" (Fig. 3) and "Pooled Diagnostic Odds Ratio (a) and SROC curve of included studies (b)" (Fig. 4). LR and DOR have been generally used to demonstrate the validity of diagnostic indicators [68]. In fact, a previous guideline clearly defined PLR > 2, NLR < 0.5, or DOR > 4 as a viable predictor, while PLR > 5, NLR < 0.2, or DOR > 10 was considered a good predictor [69]. The pooled PLR, NLR and DOR of this meta-analysis were 18.68, 0.24 and 85.65, respectively. From the results of our study, evidence of SFC on PJI was more favorable but not yet strong. Therefore, there is still much work to be done in the future, and more time and evidence are needed to prove that SFC is a reliable detection method and of great value in PJI diagnosis.

Admittedly, this meta-analysis has certain limitations. First, there is still no established gold standard for the detection of PJI. Individual studies used different reference standards, which may affect the diagnostic accuracy of a test method. Second, there was significant heterogeneity among the included studies because they were completed in different institutions and used different test methods or sample sources. Third, only 23 studies recorded the threshold, and the optimal diagnostic threshold of SFC could not be calculated due to incomplete original data. Finally, nearly half of the included studies were retrospective studies, which may reduce the strength of our study conclusions.

## Conclusion

In summary, our findings were somewhat in line with a previous meta-analysis published by Zhai et al. Based on the results of the current study, we conclude that the evidence of SFC on PJI is more favorable but not yet strong because the results from recent publications (until 2020 in our study) are still inconsistent. Therefore, improvement of the diagnostic accuracy of SFC is still necessary, and the diagnosis of PJI continues to warrant a multiplex approach before and during a revision procedure. Additionally, another meta-analysis on this topic should be conducted after years.

## Data Availability

The datasets used and/or analyzed during this study are not publicly available due to feasibility, but are available from the corresponding authors on reasonable request.

## Appendix: Search strategy

**Pubmed**

**#1**"Prosthesis-Related Infections"[Mesh]

**#2**

(((((((((Prosthesis Related Infections[Title/Abstract]) OR (Prosthesis Related Infection[Title/Abstract])) OR (Infection, Prosthesis Related[Title/Abstract])) OR (Related Infection, Prosthesis[Title/Abstract])) OR (Related Infections, Prosthesis[Title/Abstract])) OR (Prosthesis-Related Infection[Title/Abstract])) OR (Infections, Prosthesis-Related[Title/Abstract])) OR (Periprosthetic Joint Infection[Title/Abstract])) OR (PJI[Title/Abstract])) OR (periprosthetic infection[Title/Abstract])

**#3 #1 OR #2**

**#4**: "Ultrasonics"[Mesh]

**#5**

((Ultrasonic[Title/Abstract]) OR (Sonication[Title/Abstract])) OR (Sonications[Title/Abstract])

**#6 #4 OR #5**

**#7**

"sensitiv*"[Title/Abstract] OR "sensitivity and specificity"[MeSH Terms] OR ("predictive"[Title/Abstract] AND "value*"[Title/Abstract]) OR ("predictive value of tests"[MeSH Terms] OR ("predictive"[All Fields] AND "value"[All Fields] AND "tests"[All Fields]) OR "predictive value of tests"[All Fields]) OR "accuracy*"[Title/Abstract]

**#8 #3 AND #6 AND #7**

**Embase**

**#1** 'prosthesis related' AND ('infections'/exp OR infections)

**#2**

'prosthesis related infections':ab,ti OR 'prosthesis related infection':ab,ti OR 'infection, prosthesis related':ab,ti OR 'related infection, prosthesis':ab,ti OR 'related infections, prosthesis':ab,ti OR 'prosthesis-related infection':ab,ti OR 'infections and infestations':ab,ti OR 'periprosthetic joint infection':ab,ti OR 'pji':ab,ti OR 'periprosthetic infection':ab,ti

**#3: #1 OR #2**

**#4** 'ultrasound'/exp

**#5** 'ultrasonic':ab,ti OR 'sonication':ab,ti OR 'sonications':ab,ti

**#6: #4 OR #5**

**#7** 'sensitiv':ab,ti OR 'sensitivity and specificity':ab,ti OR 'value':ab,ti OR 'predictive':ab,ti OR 'predictive value of tests':ab,ti OR 'accuracy':ab,ti

**#8 #3 AND #6 AND #7**

**Cochrane**

**#1**

MeSH descriptor: [Prosthesis-Related Infections] explode all trees

**#2**

(Prosthesis Related Infections):ti,ab,kw OR (Prosthesis Related Infection):ti,ab,kw OR (Infection, Prosthesis Related):ti,ab,kw OR (Related Infection, Prosthesis):ti,ab,kw OR (Related Infections, Prosthesis):ti,ab,kw OR (Prosthesis-Related Infection):ti,ab,kw OR (Infections, Prosthesis-Related):ti,ab,kw OR (Periprosthetic Joint Infection):ti,ab,kw OR (PJI):ti,ab,kw OR (periprosthetic infection):ti,ab,kw

**#3: #1 OR #2**

**#4**

MeSH descriptor: [Ultrasonics] explode all trees

**#5**

(Ultrasonic):ti,ab,kw OR (Sonication):ti,ab,kw OR (Sonications):ti,ab,kw

**#6: #4 OR #5**

**#7** (sensitiv):ti,ab,kw OR (sensitivity and specificity):ti,ab,kw OR (value):ti,ab,kw OR (predictive):ti,ab,kw OR (predictive value of tests):ti,ab,kw OR (accuracy):ti,ab,kw

**#8 #3 AND #6 AND #7**

**Web of Science**

**#1**

TS = (Prosthesis-Related Infections or Prosthesis Related Infections or Prosthesis Related Infection or Infection, Prosthesis Related or Related Infection, Prosthesis or Related Infections, Prosthesis or Prosthesis-Related Infection or Infections, Prosthesis-Related or Periprosthetic Joint Infection or PJI or periprosthetic infection)

**#2**

TS = (Ultrasonics or Ultrasonic or Sonication or Sonications)

**#3**

TS = (sensitiv or sensitivity and specificity or value or predictive or predictive value of tests or accuracy)

**#4 #1 AND #2 AND #3**

## Abbreviations

PJI: Periprosthetic joint infection
TJA: Total joint arthroplasty
IDSA: Infectious Diseases Society of America
MSIS: Musculoskeletal Infection Society
ICM: International Consensus Meeting
SFC: Sonication fluid culture
PTC: Periprosthetic tissue cultures
TP: True positive
FP: False positive
FN: True negative
TN: False negative
PLR: Positive likelihood ratio
NLR: Negative likelihood ratio
DOR: Diagnostic odds ratio
AUC: Area under the curve
QUADAS-2: Quality Assessment of Diagnostic Accuracy Studies-2
CFU: Colony-forming unit
